# Development of mRNA–lipid nanoparticle intrabodies against rickettsial infection

**DOI:** 10.1186/s12929-025-01171-5

**Published:** 2025-08-12

**Authors:** Qi Yan, Nan Duan, Mingqun Lin, Wenqing Zhang, Stephen Denton, Yichen Zhong, Yizhou Dong, Yasuko Rikihisa

**Affiliations:** 1 Department of Veterinary Biosciences, College of Veterinary Medicine, The Ohio State University, 1925 Coffey Road, Columbus, OH 43210 USA; 2 Icahn Genomics Institute, Icahn School of Medicine at Mount Sinai, New York, NY 10029 USA

**Keywords:** *Ehrlichia chaffeensis*, Rickettsia, Anti–Etf-2, Intrabody, Endosome, RAB5, RABGAP5, Etf-2, T4SS effector, VHH, mRNA-LNP

## Abstract

**Background:**

Rickettsiosis is among the deadliest vector-borne infectious diseases worldwide, in part because rickettsiae replicate within human cells, where antibodies and most drugs cannot effectively reach this obligatory intracellular pathogen. *Ehrlichia chaffeensis*, an emerging rickettsia, is the causative agent of human monocytic ehrlichiosis. We therefore aim to generate intrabodies (IBs), the variable domain of heavy chain of heavy-chain-only antibodies (VHHs) that bind intracellular bacterial proteins to inhibit *E. chaffeensis* infection.

**Methods:**

*E. chaffeensis* replicates in membrane-bound vacuoles resembling early endosomes in human monocytes/macrophages. The type IV secretion system effector *Ehrlichia* translocated factor-2 (Etf-2) directly binds to RAB5-GTP on *E. chaffeensis-*containing vacuoles. Consequently, Etf-2 hinders the engagement of RAB5 GTPase-activating protein with RAB5-GTP, delays maturation of *Ehrlichia* vacuoles to late endosomes, thus facilitates infection. As C-terminal half of Etf-2 (Etf-2C) binds RAB5-GTP, a random synthetic library of VHHs was screened for binding to Etf-2C, and for inhibition of Etf-2 binding to RAB5 in human cells when expressed intracellularly (IBs). Positive IBs were tested for inhibition of Etf-2 functions and *E. chaffeensis* infection, and lipid nanoparticles-encapsulated mRNAs (mRNAs-LNP) platform was used to deliver IBs in vitro and in mice.

**Results:**

We have identified two distinct IBs that inhibit Etf-2 binding to RAB5 and Etf-2 functions in vitro. Synthesized mRNA-LNP encoding anti-Etf-2 IBs significantly inhibited *E. chaffeensis* infection in cell cultures and in a mouse model.

**Conclusions:**

The results demonstrate the feasibility of mRNA-LNP encoding IBs as intracellular probes and a precision therapy addressing underlying cause of obligatory intracellular infection.

**Supplementary Information:**

The online version contains supplementary material available at 10.1186/s12929-025-01171-5.

## Background

Rickettsial diseases, including anaplasmosis and ehrlichiosis, are greatly increasing in worldwide prevalence [5]. An emerging infectious disease with serious health impacts called human monocytic ehrlichiosis (HME), which is caused by *Ehrlichia chaffeensis*, is a typical example [28, 29]. HME is a severe flu-like illness often accompanied by hepatitis with a high rate of hospitalization (40–63%) [1, 16, 38]. Life-threatening complications such as renal failure, adult respiratory distress syndrome, meningoencephalitis, multi-system organ failure, and toxic shock occur in a substantial portion of hospitalized patients, with a case fatality rate of 3% [28, 38]. Although doxycycline is effective, HME can be fatal when antibiotic treatment is delayed, particularly for persons aged > 60 years and for immunocompromised individuals [1, 28, 38]. No FDA-approved vaccine exists for any rickettsial infection including *E. chaffeensis,* underscoring the importance of developing new prophylactic and therapeutic approaches for rickettsial infections [15].

*E. chaffeensis* replicates in membrane-bound vacuoles which retain a regulator of endosomal traffic, the small GTPase RAB5 to avoid routing to host-cell phagolysosomes [21, 40]. The bacterial type IV secretion system (T4SS) secretes bacterial effector molecules into eukaryotic target cells across the eukaryotic cell membrane; these molecules ultimately promote bacterial infection and disease[12]. The *Ehrlichia* T4SS effector Etf (*Ehrlichia* translocated factor)-2 is a unique RAB5-GTP–binding protein that impedes fusion of *Ehrlichia* vacuoles with lysosomes by blocking the interaction between RAB5 GTPase-activating protein (RABGAP5) and RAB5-GTP [40]. Thus RAB5-GTP persists on *E. chaffeensis*–containing vacuoles, which prevents lysosomal fusion [40]. As knockdown of Etf-2 in *E. chaffeensis* significantly inhibits *E. chaffeensis* infection [40], Etf-2 represents an ideal intracellular target for blocking infection.

The variable domain of the heavy chain of heavy-chain-only antibodies (VHHs) of camelids is small (11–15 kDa) but retains full antigen specificity and binding affinity [13]. Cloned VHHs that are expressed and can function within mammalian cells are called intrabodies (IBs) [6] and can be directed against intracellular antigens. Separately, mRNA-based technologies offer well-known and validated advantages, such as the relative simplicity of these approaches, the ability to rapidly engineer mRNA to encode proteins of interest, the absence of a requirement for genomic integration or protein purification, and the ability to rapidly and efficiently produce such proteins, all of which have laid the groundwork for a wide range of applications [17]. To ensure proper function in vivo, however, mRNA requires effective and stable delivery systems to protect it from degradation while allowing its uptake and release. For this, lipid nanoparticle (LNP)-encapsulated mRNA (mRNA-LNP) has become a breakthrough vaccine delivery platform because of its effectiveness at preventing infection by the SARS-CoV-2 virus [14]. An LNP-based mRNA delivery platform has also emerged as a new delivery method for therapeutic agents to prevent and treat various diseases. Here, we demonstrate the identification of two distinct anti–Etf-2 IBs by sequential screening of a random synthetic VHH library for Etf-2 binding and for neutralization of intracellular Etf-2 function. The two mRNA-LNP anti–Etf-2 IBs significantly inhibited *E. chaffeensis* infection in vitro and in vivo, suggesting the utility of this approach for discovering IBs to reduce the severity and prevalence of rickettsial diseases.

## Methods

### Bacteria and cell culture

The *E. chaffeensis* Arkansas strain [8] was obtained from the Centers for Disease Control and Prevention (Atlanta, GA) and cultured in the nonadherent human acute leukemia cell line THP-1 cells (ATCC, Manassas, VA) in RPMI 1640 medium (Mediatech, Manassas, VA) supplemented with 8% fetal bovine serum (FBS; Atlanta Biologicals, Lawrenceville, GA) and 2 mM l-glutamine (GIBCO, Waltham, MA). To study IBs protein expression and localization in human cells, HeLa cells (ATCC) were used. As transfection of THP-1 cells is difficult and HeLa cells cannot be infected with *E. chaffeensis,* human embryonic kidney HEK293 and HEK293T cells (ATCC) that can be easily transfected and readily infected with *E. chaffeensis* [21, 40] were used to study protein functions and protein–protein interactions in host cells. Monkey endothelial RF/6A cells (ATCC), which are thinly-spreading adherent non-phagocytic cells, have higher transfection efficiencies than THP-1 cells, and can be readily infected with *E. chaffeensis*, are used for unambiguous subcellular localization studies of bacteria-containing vacuoles with endosomes or latex beads [22]. HeLa, HEK293 or HEK293T cells were cultured in Dulbecco’s minimal essential medium (DMEM; Mediatech) supplemented with 5% FBS and 2 mM l-glutamine. RF/6A cells were cultured in advanced minimal essential medium (AMEM; GIBCO) supplemented with 8% FBS and 2 mM l-glutamine. All cultures were incubated at 37 °C under 5% CO_2_ in a humidified atmosphere.

### Antibodies

The following antibodies were used in this study: mouse monoclonal anti-hemagglutinin (HA) (BioLegend, San Diego, CA), rabbit anti-HA (Cell Signaling, Danvers, MA), rabbit anti-mCherry (Invitrogen, Carlsbad, CA), mouse monoclonal anti-green fluorescent protein (GFP) and rabbit anti-RAB5 (Santa Cruz Biotechnology, Dallas, TX), rabbit anti-tubulin and mouse anti-actin (Sigma-Aldrich, St. Louis, MO), Alexa Fluor (AF)350-conjugated goat anti–mouse IgG and AF647-conjugated goat anti–rabbit IgG (Invitrogen), and horseradish peroxidase (HRP)-conjugated goat anti–mouse or –rabbit IgG (SeraCare, Milford, MA).

### Purification and biotinylation of rEtf-2C

The gene encoding Etf-2C (aa 152–264) was fused with a C-terminal 6 × His-tag and cloned into pET-33b( +) vector. Recombinant Etf-2C was expressed in *Escherichia coli* BL21 (DE3) (NEB, Ipswich, MA). rEtf-2C expression was induced by adding 1 mM isopropyl-1-thio-β-d-galactopyranoside when the cells reached an optical density at 600 nm of 0.6, and the cells were continuously cultured at 30 °C for 5 h. The *E. coli* cells were harvested by centrifugation at 5,000 × *g* for 15 min and lysed by sonication in lysis buffer (50 mM sodium phosphate, pH 7.4; 0.3 M NaCl; 10% glycerol; 0.05% NaDoc; 0.05% CHAPS; 20 mM imidazole; and 1 mM PMSF) and then centrifuged at 18,000 × *g* for 20 min at 4 °C [20]. The resulting supernatant was used to purify soluble protein by affinity chromatography using HisPur Cobalt resin (Thermo Fisher Scientific, Waltham, MA). Biotinylation of rEtf-2C was performed via EZ-Link NHS-Biotin Reagents (Thermo Fisher Scientific) and biotinylated rEtf-2C was then applied to a Superdex 75 Increase 10/300 GL column (Cytiva, Marlborough MA) in Dulbecco’s modified phosphate-buffered saline (PBS: 8 mM Na_2_HPO_4_, 1.47 mM KH_2_PO_4_, 2.67 mM KCl, 137.9 mM NaCl, pH 7.4).

### Screening the synthetic VHH library

The hs2dAb Phage Display library (Hybrigenics Services SAS, Paris, France), which presents 3 × 10^9^ synthetic humanized VHHs, was screened to identify VHHs that recognize Etf-2C. Etf-2C–biotin and His-SNAP-Halo-Biotin (Hybrigenics) were separately bound to Streptavidin Magnetic Beads (Dynabeads M-280 Streptavidin, Invitrogen) with a 50 nM final concentration of biotinylated protein. First, the His-SNAP-Halo was used to deplete nonspecific binders from the library. Then, the unbound VHH-expressing phages were incubated with the Etf-2C–biotin beads to enrich for phages with specificity for Etf-2C. From this first round of screening, 2.1 × 10^6^ phages were collected and used to create a yeast prey library (complexity, 2.2 × 10^6^ yeast cells), which was used to screen for Etf-2C–binding VHHs with a LexA-based Yeast two-hybrid (Y2H) system [11]. Using LexA–Etf-2C as the bait, we isolated 190 positive clones from among 16 million interactions. Sequencing these clones indicated the presence of 15 distinct VHH clones.

To assess mammalian cell expression and intracellular binding to Etf-2C, the 10 selected VHHs were individually subcloned into the mammalian vector pmCherry (Addgene, Watertown, MA) which resulted in the fusion of the VHH to mCherry fluorescent protein for cytoplasmic expression. Etf-2C was subcloned into the mammalian vector pEM15 to create Etf-2C–GFP with a membrane localization signal (neuraminidase target signal). HeLa cells were co-transfected (JetPEI DNA transfection from Polyplus, New York, NY) with an mCherry-VHH and Etf-2C–GFP, and the binding of the VHH to Etf-2C was assessed by fluorescence microscopy at 1 day post transfection (dpt).

### Screening anti–Etf-2C IBs for inhibition of Etf-2–GFP binding to endogenous RAB5

Exponentially growing HEK293T cells (2 × 10^6^ cells) were suspended in 100 μl Opti-MEM (GIBCO) and co-transfected with plasmids expressing Etf-2 (full-length)–GFP [40] and each of 10 mCherry-IBs that bind Etf-2C or mCherry alone as a negative control by electroporation at 100 V and 1,000 μF using the Gene Pulser Xcell System (Bio-Rad, Hercules, CA); electroporated cells were then plated in T25 flasks. The resulting transfected cells were harvested at 2 dpt, and lysed in ice-cold lysis buffer (25 mM Tris, pH 7.6; 150 mM NaCl; 1% NP-40; protease inhibitor cocktail [1:100]) by end-to-end rotation at 20 rpm for 15 min at 4 °C. Proteins in the lysates were immunoprecipitated with anti-GFP nanobody affinity gel (BioLegend, San Diego, CA) and analyzed by western blotting (WB) with mouse anti-GFP (1:500), rabbit anti-tubulin (1:1,000), rabbit anti-mCherry (1:1,000), and rabbit anti-RAB5 (1:1,000), followed by HRP-conjugated secondary antibodies (1:2,000). Immunoreactive bands were visualized with Pierce ECL WB Substrate (Thermo Fisher), and images were captured and quantified with an Amersham AI680QC gel documentation system (GE Healthcare, Marlborough, MA).

### Endosome and *E. chaffeensis* vacuole localization analysis of anti–Etf-2C IBs and Etf-2

RF/6A cells were triple-transfected with plasmids encoding Etf-2–GFP, HA-RAB5^CA^ (constitutively active RAB5) [40], and mCherry-A44, -A123, or -A171 by electroporation and cultured on coverslips in the wells of a 12-well plate. Subcellular localization was determined at 2 dpt. Cells were fixed in 4% paraformaldehyde (PFA) and incubated with rabbit anti-HA in PGS (PBS supplemented with 0.5% BSA [Sigma], 0.1% gelatin [Sigma], and 0.1% saponin [Sigma]) followed by AF647-conjugated goat anti–rabbit IgG in PGS.

For time-course analysis of endosome maturation, Flash Red (near-infrared) latex beads (1 µm; Bangs Laboratories, Fishers, IN) were coated with recombinant C terminus of the *E. chaffeensis* invasin EtpE, Entry-triggering protein of *Ehrlichia* (EtpE-C) protein (EtpE-C–Flash Red–beads), as described [26]. RF/6A cells cultured on coverslips in a 24-well plate were triple-transfected with plasmids encoding Etf-2–GFP; mCherry-A44, -A123, or -A171; and HA-RAB5 (wild type) with Lipofectamine 3000 (Invitrogen) for 2 d. Freshly prepared EtpE-C–Flash Red–beads were added to the wells of a 24-well plate (~ 5 × 10^6^ beads per well). After incubation at 37 °C with 5% CO_2_ in a humidified atmosphere for 30–120 min, uninternalized beads were removed by washing with PBS. Cells were then fixed in 4% PFA and incubated with mouse monoclonal anti-HA followed by AF350-conjugated goat anti–mouse IgG in PGS.

For *E. chaffeensis* vacuole localization analysis, *Ehrlichia*-infected RF/6A cells on coverslips in the wells of a 24-well plate at 1 dpi were co-transfected with plasmids encoding Etf-2–GFP and mCherry-A44, -A123, or -A171 with Lipofectamine 3000. Cells were fixed in 4% PFA at 2 dpt. DAPI (Sigma) was used to label DNA (in the host cell nucleus and to label *Ehrlichia*).

### Image acquisition and analysis

Fluorescence images with overlaid differential interference contrast (DIC) images were captured with a DeltaVision PersonalDV deconvolution microscope system equipped with a 4-color filter set (DAPI-FITC-TRITC-Cy5) (GE Healthcare Life Sciences, Marlborough, MA). TRITC filter was used for mCherry (λ_EX_/λ_EM_ = 587 nm/610 nm), and Cy5 filter was used for Flash Red (λ_EX_/λ_EM_ = 660 nm/690 nm). Colocalization analysis was performed on a single *z*-section by assessing fluorescence signals for > 100 vacuoles, phagosomes, or vesicles per cell in 20–30 cells per experiment to obtain percentage colocalization of RAB5^CA^ endosomes, endosomes containing beads, or *E. chaffeensis* vacuoles with various markers including anti–Etf-2C IBs, Etf-2–GFP, and HA-RAB5.

### Effect of IB A44, A123, and A171 on *E. chaffeensis* infection

HA-tagged IBs A123, A44, and A171 were constructed from the plasmids encoding mCherry-IBs by two-step PCR with overlapping primers and were subsequently cloned into pEGFP-N1 (Takara, Mountain View, CA) by replacing the GFP-tag with the HA-tag (Table S1). HEK293T cells were co-transfected with HA-A44, HA-A123, HA-A44 plus HA-A123, or HA-A171 plasmid or were sham-transfected by electroporation. Transfected cells were infected with freshly isolated *E. chaffeensis* at a multiplicity of infection (MOI) of 30 at 1 dpt. Bacteria that were not internalized were removed at 1 dpi, and cells were harvested at 2 dpi. Bacterial and host gene expression was estimated by RT-qPCR (see below).

### Anti–Etf-2C epitope analysis

HEK293T cells were co-electroporated with Etf-2–GFP or one of its mutated forms Etf-2^R188A^ (Etf-2^RA^)–GFP, Etf-2^Q245A^ (Etf-2^QA^)–GFP, or Etf-2^R188A/Q245A^ (Etf-2^DM^)–GFP[40], in addition to mCherry-A44, -A123, or -A171. Cells were lysed at 2 dpt as described above, and the resulting lysates were immunoprecipitated with anti-GFP with nanobody affinity gel and subjected to WB with anti-GFP, anti-mCherry, and anti-Actin (1:1,000 dilution each).

### Surface plasmon resonance (SPR) analysis

A44, which was codon optimized for *E. coli* expression, and A123 and A171 VHHs were cloned into the pMECS vector (Table S1). The plasmids were purified from TG1 *E. coli* and transformed into WK6 *E. coli*, a non-suppressor strain (*supE−*) (both strains from ATCC, Manassas, VA). The VHHs, each of which contains the periplasmic localization sequence PelB signal peptide at its N terminus, were expressed and purified from the periplasm as described [41]. The supernatant containing the VHHs, which also contain a C-terminal HA-tag followed by a 6 × His-tag, was affinity-purified with cobalt resin (Thermo Scientific) as described [41]. These purified VHHs were used for SPR analysis with the OpenSPR system (Nicoya Lifesciences, Ontario, Canada). Briefly, biotinylated rEtf-2C proteins were immobilized onto a streptavidin sensor chip (Nicoya Lifesciences) in PBS running buffer (PBS containing 0.05% Tween 20). Serial dilutions of purified A123, A44, or A171 were slowly flowed over the sensor chip at a rate of 30 µL/min with a contact time of 270 s and dissociation time of 330 s in PBS running buffer. Between each IB injection, the sensor chip was regenerated by injecting 10 mM glycine buffer for three times until a stable baseline is obtained. Data were analyzed and binding kinetics were obtained using the TraceDrawer program (Ridgeview Instruments, Uppsala, SWEDEN).

### Formulation of mRNA-LNPs

The linear double-stranded DNA sequences of the IBs of interest were codon-optimized for mammalian expression and obtained from Integrated DNA Technologies (Coralville, IA). These gene fragments were inserted into the plasmid pUC19 via Gibson assembly (NEB) (Figure S1A) and amplified to generate the DNA template for in vitro transcription. Then, IB mRNAs were prepared through in vitro transcription (NEB) followed by purification using RNA Clean & Concentrator (Zymo Research, Irvine, CA). Purified in vitro–transcribed mRNAs were treated with vaccinia capping enzyme (NEB) to generate Cap-0 at the 5'-end, and then with 2'-*O*-methyltransferase (NEB) to generate Cap-1, which reduces the cellular innate immune response [7, 32]. The capped mRNAs were further purified (Zymo), and their concentrations were measured with a NanoDrop 2000 Spectrophotometer (Thermo Fisher Scientific). mRNA-LNPs were prepared by rapidly mixing mRNA in citrate buffer (pH 4.0) with lipid solutions dissolved in ethanol using Rapid Nanomedicine System INano L + (Micro&Nano Biologics Technology Ltd., Shanghai, China) as described [10]. The encapsulation efficiency of LNPs was ~ 90% (Figure S1B), as determined by the RiboGreen assay (Quant-iT RiboGreen RNA Reagent, Thermo Fisher Scientific) and by quantification using Cytation 5 (BioTek, Winooski, VT).

### In vitro and in vivo delivery of IB-encoding mRNA-LNPs and *E. chaffeensis* infection

HEK293 cells (2 × 10^6^ cells) were transfected with 2 μg of mRNA-LNPs encoding A44, A123, or A171 or with an equal volume of PBS as a negative control for 6 h. The cells were then infected with host cell–free *E. chaffeensis* at an MOI of 20 for 30 h, followed by preservation in RNAlater buffer (Qiagen) at –80 °C for RNA extraction for RT-qPCR analysis.

To deliver IB mRNA-LNPs in vivo and to examine their effects on *Ehrlichia* infection, ICR mice (five mice per group; Inotiv, West Lafayette, IN) were intraperitoneally inoculated with *E. chaffeensis* cultured in THP-1 cells (~ 2 × 10^5^ bacteria per mouse), which were preincubated with IB mRNA-LNPs (A44, A123, or A171) or with PBS for 12 h. As *E. chaffeensis* infects blood monocytes and macrophages, intravenous injection of LNP-mRNAs allows immediate delivery to the bloodstream and rapid systemic distribution to infected cells [36]. At 1 and 3 dpi, mice were intravenously inoculated through the retro-orbital venous plexus with IB mRNA-LNPs (10 μg in a 100-μl volume of PBS per mouse) or with an equal amount of PBS as a negative control. Mice were monitored daily for body weight and clinical signs (squinting eyes, anorexia, and inactivity) after infection with *E. chaffeensis*. At 5 dpi, mice were euthanized by CO_2_ inhalation followed by cervical dislocation. Blood, liver, and spleen samples were harvested and stored at –20 °C for DNA extraction or preserved in RNAlater buffer at –80 °C for RNA extraction.

### qPCR and RT-qPCR analysis

DNA and RNA samples were purified using the DNeasy Blood & Tissue kit (Qiagen, Germantown, MD) and RNeasy Mini kit (Qiagen), respectively. cDNA was synthesized from 1 µg RNA using the Maxima H minus First Strand cDNA synthesis kit with random hexamer primers (Thermo Fisher Scientific). The qPCR and RT-qPCR analyses were performed with primers targeting the *E. chaffeensis* 16S rRNA gene and mouse *Gapdh* [24] (Table S1) using Maxima SYBR Green/ROX Master Mix (Thermo Fisher Scientific) with an AriaMx Real-time PCR system (Agilent, Santa Clara, CA). The bacterial inoculum was quantified by qPCR using *Ehrlichia* 16S rDNA cloned into plasmid pUC19 as the standards [33].

### Statistical analysis

All statistical analyses were performed with one-way or two-way analysis of variance (ANOVA) followed by Dunnett’s test using Prism 9 software (GraphPad, Boston, MA). *P* < 0.05 was considered statistically significant.

## Results

### Anti-Etf-2 VHH clones that block the interaction between Etf-2–GFP and endogenous RAB5

**Fig. 1:**
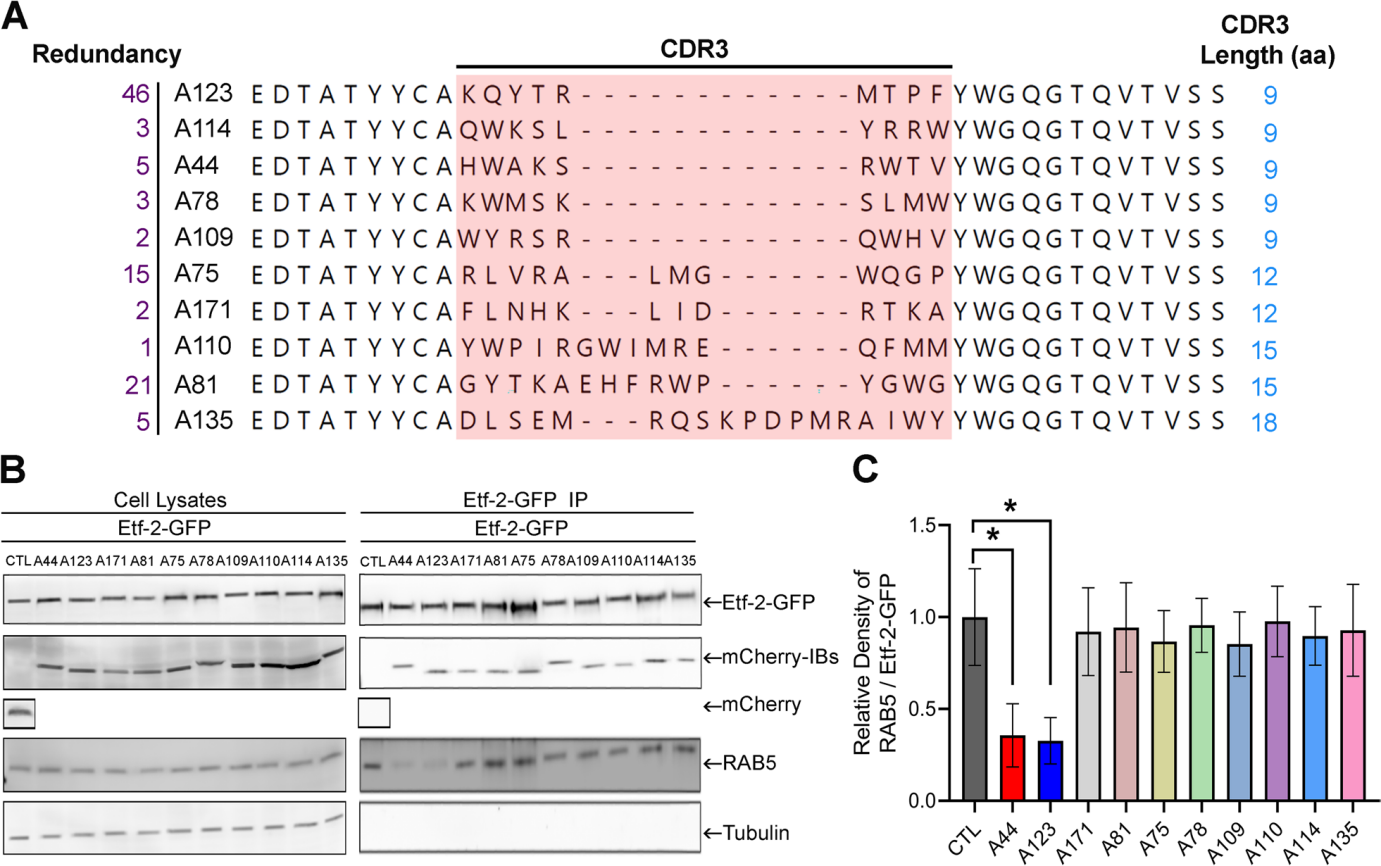
Etf-2C–binding VHH clones that block the interaction between Etf-2–GFP and endogenous RAB5. **A** Amino acid sequence alignment of anti–Etf-2C VHHs. CDR3 sequences are highlighted with red shading, redundancy among the Y2H clones is shown on the left, and the number of residues in each CDR3 is shown on the right. **B** HEK293T cells were co-transfected with plasmids encoding Etf-2–GFP and each of 10 mCherry-IBs or mCherry alone (CTL), lysed at 2 dpt, immunoprecipitated with anti-GFP affinity gel, and analyzed by WB with antibodies against GFP, mCherry, RAB5, and Tubulin. IP, immunoprecipitate. **C** Quantification of the relative density of RAB5 bound to Etf-2–GFP from blots as in (**B**). Data are presented as the mean ± standard deviation (SD) from three independent experiments. *Significantly different as determined by one-way ANOVA followed by Dunnett’s test (CTL vs A44, *P* = 0.0053; CTL vs A123, *P* = 0.0034)

The C-terminal half of Etf-2 (Etf-2C, aa 152–264) directly binds RAB5-GTP [40]. Thus, we screened a synthetic humanized random VHH M13 phage display library hsd2Ab by affinity purification using recombinant Etf-2C (rEtf-2C)-Biotin bound to Streptavidin Magnetic Beads. This resulted in ~ 1,000-fold enrichment of Etf-2C–binding phages (2.1 × 10^6^ phages). Next, we used a Y2H system to screen the prey library of VHHs encoded in the enriched Etf-2C–binding phages, with Etf-2C cloned into the bait vector. Alignment of the deducted amino acid (AA) sequences of 190 positive VHH clones identified 15 distinct anti–Etf-2C VHH clones. Among them, one clone containing a predicted glycosylation site in the complementarity-determining region 3 (CDR3), which is the most variable region within VHHs, and four clones with no redundancy were excluded from further analysis, resulting in 10 distinct VHH clones (CDR3 sequences and numbers of redundancy were shown in Fig. 1A). To determine expression levels of VHHs and intracellular colocalization with Etf-2 in human cells, 8 mCherry-VHH clones with more than 2 redundancies (excluding A109 and A110, Fig. 1A) were double transfected with Etf-2C-GFP in HeLa cells. The result showed all 8 clones were highly expressed and colocalize with Etf-2C-GFP (Figure S2).

Etf-2 (full-length)–GFP binds to endogenous RAB5 [40], and *E. chaffeensis* can infect HEK293T cells, but not HeLa cells. Thus, we tested in HEK293T cells whether above 8 mCherry-VHH clones and additional two untested clones of low redundancy (A109 and A110) when expressed intracellularly (IBs) could bind Etf-2, and interfere binding of Etf-2–GFP to endogenous RAB5. Anti-GFP affinity gel pull-down assay followed by WB results showed all 10 mCherry-anti–Etf-2C IBs with distinct CDR3 sequences (Fig. 1A), but not mCherry alone, bound to Etf-2–GFP (Fig. 1B), validating our Y2H and colocalization analysis results. Nonetheless, only two IBs: A44 and A123, significantly inhibited Etf-2–GFP binding to endogenous RAB5, but not the rest of eight IBs or mCherry alone (Fig. 1B and C).

### Effects of A44 and A123 on Etf-2 localization to endosomes with constitutively active RAB5 and endosome maturation

**Fig. 2:**
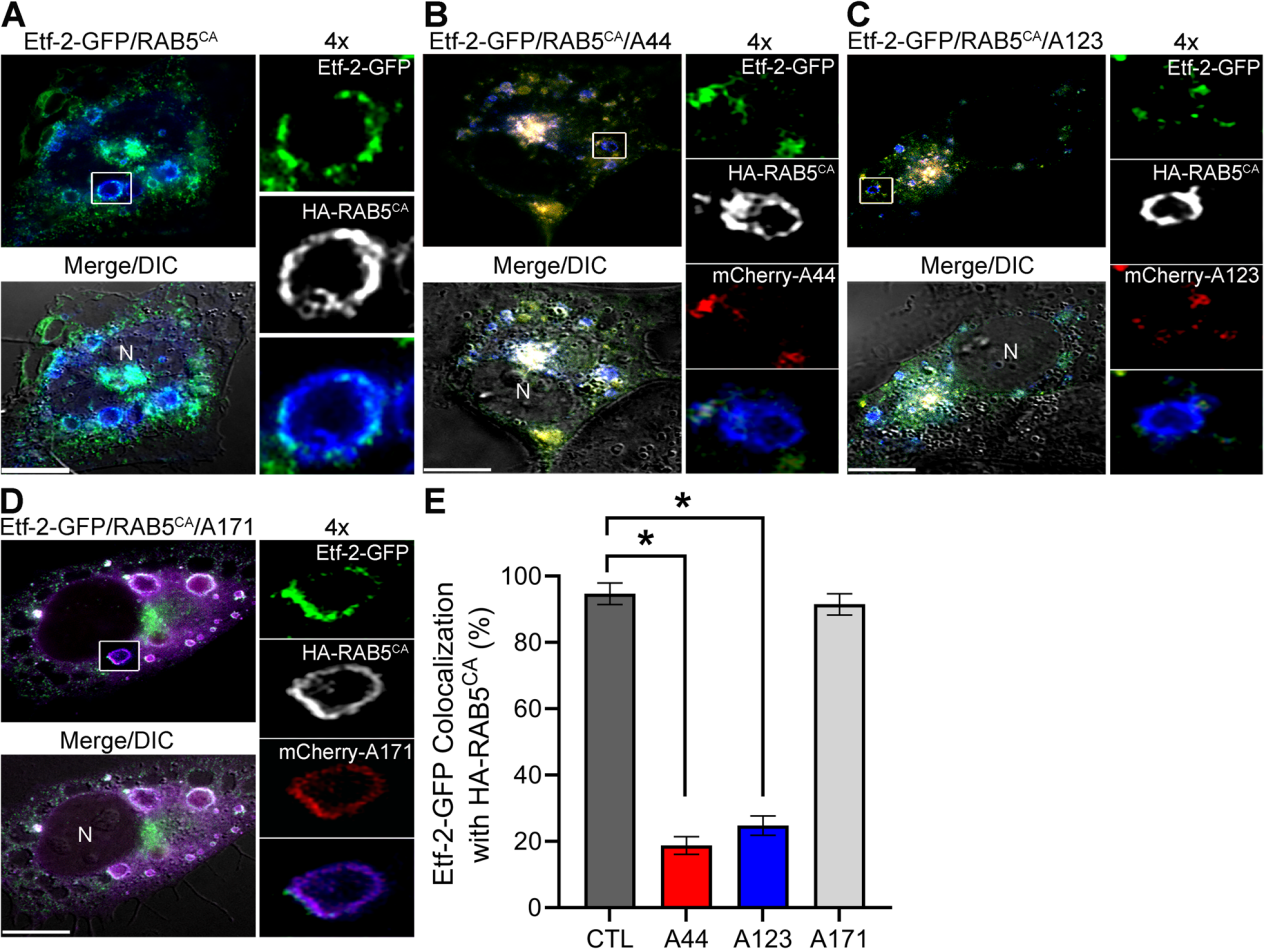
IBs A44 and A123 block Etf-2–GFP localization to early endosomal membranes. **A**–**D** RF/6A cells were triple-transfected with Etf-2–GFP, HA-RAB5^CA^, and no IB (negative CTL) (**A**), mCherry-A44 (**B**), mCherry-A123 (**C**), or mCherry-A171 (**D**). Cells were fixed at 2 dpt and immuno-stained with rabbit anti-HA, followed by AF647 (pseudocolored grey in individual channels, or shown as blue in merged channels) goat anti–rabbit IgG. Merge/DIC, the fluorescence image was merged with the DIC image; N, nucleus. Each boxed area is enlarged 4 × on the right. Scale bars: 10 µm. **E** Quantification of RAB5^CA^ endosomes in 30 transfected cells from each transfection. Data are presented as the mean ± SD from three independent experiments. *Significantly different as determined by one-way ANOVA followed by Dunnett’s test (CTL vs A44 or A123, *P* < 0.0001)

When constitutively active RAB5 (RAB5^CA^ or RAB5^Q79L^) is overexpressed, it leads to increased homotypic fusion of early endosomes, which results in the formation of enlarged early endosomes [34]. Etf-2–GFP localizes to early endosomes labeled with HA-tagged wild-type RAB5 (HA-RAB5^WT^) and HA-RAB5^CA^ in co-transfected cells [40]. Thus, we examined whether mCherry-A44 and mCherry-A123 inhibit Etf-2–GFP localization to HA-RAB5^CA^–labeled early endosomes in RF/6A cells by triple co-transfection. We used mCherry-A171, which did not inhibit Etf-2 binding to RAB5 (Fig. 1B), as the negative control. mCherry-A44 and -A123 did not localize to HA-RAB5^CA^ endosomes and prevented Etf-2–GFP localization to HA-RAB5^CA^ endosomes, whereas mCherry-A171 localized to HA-RAB5^CA^ endosomes but did not prevent Etf-2–GFP localization to HA-RAB5^CA^ endosomes, which was similar to the additional control without IBs (Fig. 2A-E).

**Fig. 3:**
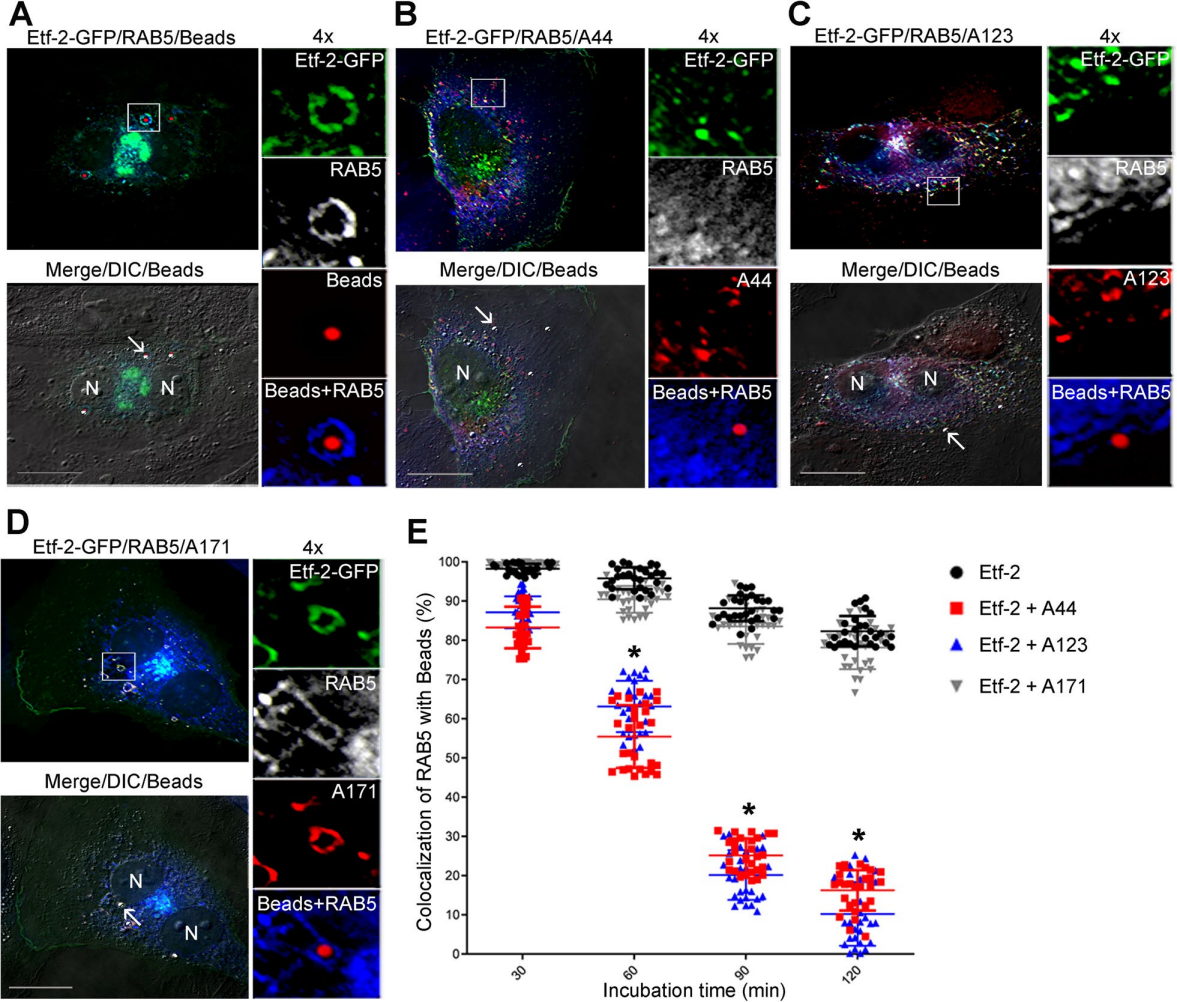
IBs 44 and A123 cause rapid dissociation of HA-RAB5 from endosomes containing EtpE-C–coated beads. RF/6A cells were triple-transfected with Etf-2–GFP; HA-RAB5; and no IB (negative CTL) (**A**), mCherry-A44 (**B**), mCherry-A123 (**C**), or mCherry-A171 (**D**) for 2 d and then were incubated for 30–120 min with EtpE-C–Flash Red–beads. Cells were harvested every 30 min after incubation with the beads. HA-RAB5 was labeled with mouse anti-HA, follow by AF350 anti–mouse IgG (shown as blue in merged images or pseudocolored grey in enlarged single-channel images). Fluorescence of EtpE-C–coated Flash Red-beads was acquired by the Cy5 infrared filter, which was pseudocolored red in panel **A** and in enlarged image panels (**B**-**D**), or pseudocolored grey in 4-channel merged images with DIC (Merge/DIC/Beads) (**B**-**D**). **A**–**D** are representative images at 60 min after bead uptake, and each boxed area is enlarged 4 × on the right. White arrows: EtpE-C–coated Flash Red-beads. Scale bars: 10 μm. **E** The scatter plots show the percent localization of RAB5 on endosomes containing EtpE-C–coated Flash Red-beads in individual transfected RF/6A cells, with the horizontal bar representing the mean value (*n* = 27 cells, which contained a total of 120–150 beads). * Significantly different as determined by a two-way ANOVA followed by Dunnett’s test at 60, 90, and 120 min (Etf-2 vs Etf-2 + A44, Etf-2 + A123, *P* < 0.0001). Representative data are shown from two independently repeated experiments

*E. chaffeensis* binds via EtpE-C to the host cell surface receptor DNase X, which induces receptor-mediated endocytosis [25, 26]. The uptake of EtpE-C–Flash Red–beads mimics the *E. chaffeensis* entry pathway into early endosomes in permissive host cells (both non-phagocytes and phagocytes) [26]. Whereas Etf-2–GFP overexpression does not inhibit receptor-mediated endocytosis of EtpE-C–Flash Red–beads by RF/6A cells or localization of RAB5 to the endosomes, it significantly delays maturation of EtpE-C–Flash Red–beads-containing endosomes into endo-lysosomes by preventing RAB5 dissociation from the endosome [40]. Thus, to determine whether A44 and A123 IBs localize to endosomes containing EtpE-C–Flash Red–beads and modulate Etf-2–mediated inhibition of endosome maturation, RF/6A cells were triple-transfected with Etf-2–GFP; HA-RAB5; and mCherry-A44, mCherry-A123, mCherry-A171, or no IB for 2 days and then were incubated with EtpE-C–Flash Red–beads. Cells were incubated with EtpE-C–Flash Red–beads for 120 min, with cell samples collected every 30 min. In RF/6A cells co-transfected with Etf-2–GFP plus mCherry-A44 or -A123, neither IB localized to endosomes containing the EtpE-C–Flash Red–beads throughout the incubation period (Fig. 3B and C). In these same cells, HA-RAB5 localized to ~ 85% of the endosomes containing the EtpE-C–Flash Red–beads at 30 min after the beads were added to the cells, but HA-RAB5 rapidly dissociated from the endosomes and was localized to very few of the bead-containing endosomes at 120 min (Fig. 3B, C, and E). In cells transfected with Etf-2–GFP and mCherry-A171 or with Etf-2–GFP alone, however, HA-RAB5 and Etf-2–GFP co-localized to almost 100% of the endosomes containing the EtpE-C–Flash Red–beads at 30 min after the beads were added to the cells, and RAB5, Etf-2–GFP, and mCherry-A171 remained localized to ~ 80% of bead-containing endosomes after a 120-min incubation (Fig. 3A, D, and E). Thus Etf-2 quickly localized with RAB5 on endosomes containing EtpE-C–Flash Red–beads in RF/6A cells, prolonged RAB5 localization on the endosomal membrane, and delayed endosomal maturation in the absence of any IBs or in the presence of A171 IB. In contrast, in the presence of A44 or A123 IBs, RAB5 progressively dissociated from the endosomes containing EtpE-C–Flash Red–beads, suggesting that these endosomes had matured into endolysosomes.

### Binding affinities of A44, A123, and A171 to Etf-2C

**Fig. 4:**
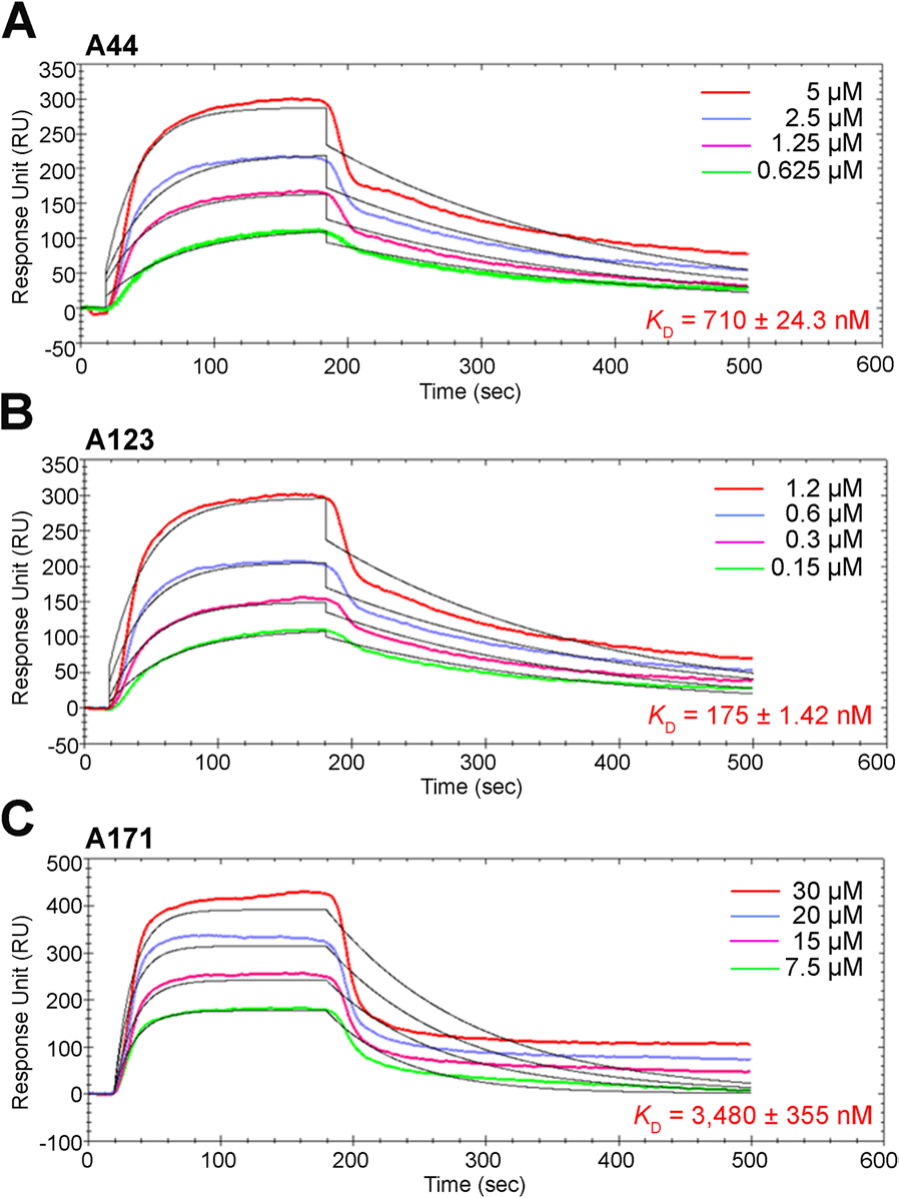
OpenSPR analyses of binding affinities of VHHs to rEtf-2C. The VHH proteins at the indicated concentrations were injected onto rEtf-2C immobilized on the sensor chip. The data indicate the binding affinities between rEtf-2C and A44 (**A**) or A123 (**B**) or the negative CTL, A171 (**C**). Colored lines were signals detected by OpenSPR, and thin black lines were fitted models generated by the TraceDrawer software. The equilibrium dissociation constant* K*_*D*_ values were calculated by the TraceDrawer program. The concentrations of IB proteins are shown in the inset of each panel

Although all three IBs (A44, A123, and A171) bound to Etf-2C, only A44 and A123 inhibited Etf-2C binding/colocalization to RAB5 regardless of the tag (mCherry vs HA) of IBs, the type of RAB5 (endogenous vs transfected HA-RAB5^CA^ or HA-RAB5), or the type of host cells (HEK293T vs RF/6A) (Figs. 1, 2, 3). Therefore, we determined the binding affinities of these three VHHs to rEtf-2C using OpenSPR to measure the kinetic constant *K*_D_, with lower *K*_D_ values indicating relatively higher binding affinities [42]. Both A44 and A123 had a specific and higher binding affinity to rEtf-2C (*K*_D_ = 710 ± 24.3 and 175 ± 1.42 nM, respectively) than did A171 (*K*_D_ = 3,480 ± 355 nM) (Fig. 4), suggesting that a low *K*_D_ value is one of the requirements for blocking Etf-2C binding to RAB5.

### A44 and A123 effects on Etf-2–GFP localization to *E. chaffeensis* vacuoles and infection

**Fig. 5:**
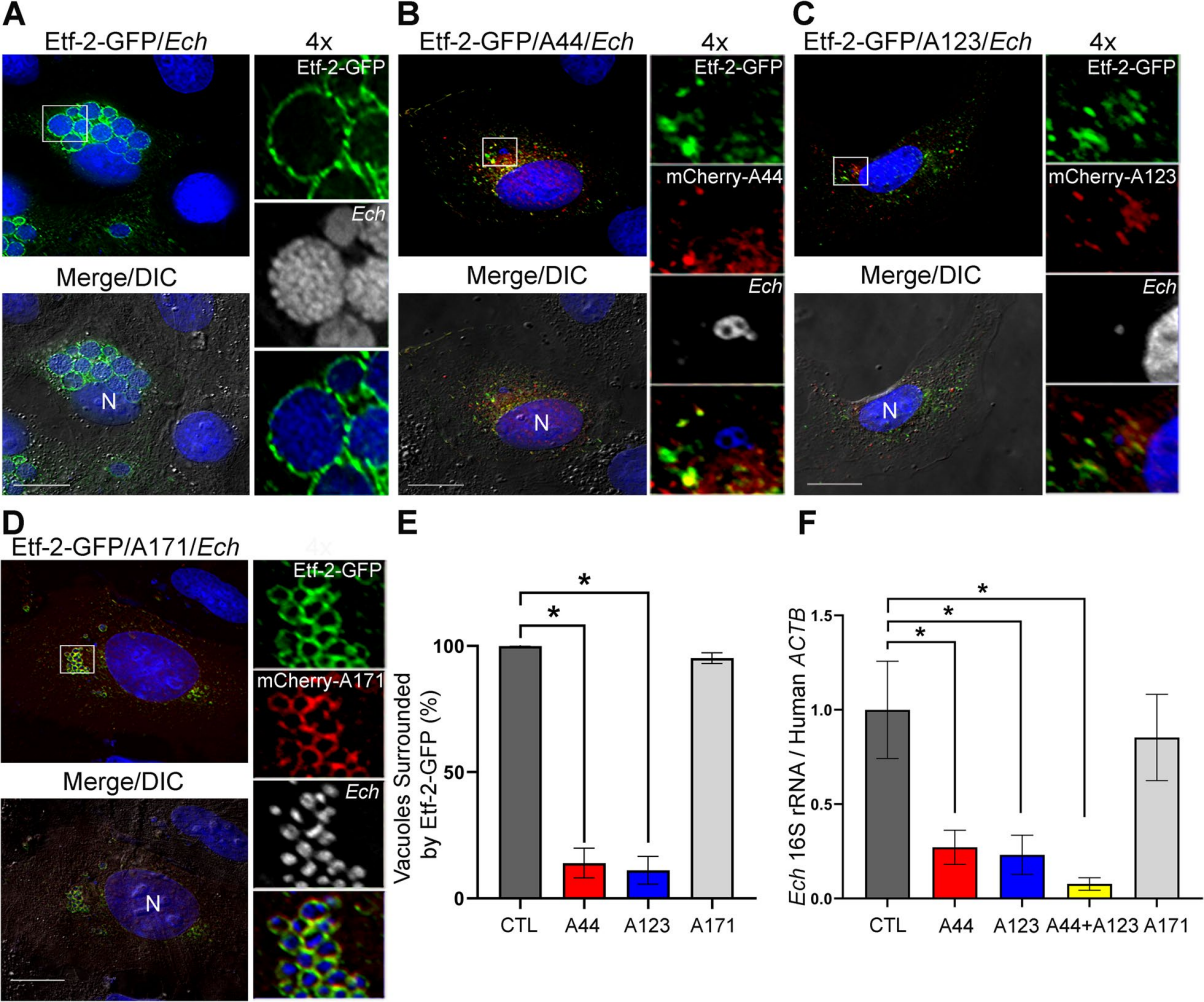
IBs A44 and A123 block Etf-2–GFP localization to *Ehrlichia* vacuoles and inhibit infection. **A**–**D** *E. chaffeensis*–infected RF/6A cells at 1 dpi were transfected with Etf-2–GFP only (CTL) (A), Etf-2–GFP and mCherry-A44 (**B**), Etf-2–GFP and mCherry-A123 (**C**), or Etf-2–GFP and mCherry-A171 (**D**). At 2 dpt (3 dpi), cells were fixed and stained with DAPI (shown as blue in merged channels, and pseudocolored grey in individual channels). Merge/DIC, the fluorescence image was merged with the DIC image; N, nucleus; *Ech, E. chaffeensis*. Each boxed area is enlarged 4 × on the right. Scale bars: 10 µm. **E** Quantification of the localization of Etf-2 to 150 *Ehrlichia* vacuoles in 30–50 transfected cells. Data are presented as the mean ± SD from three independent experiments. *Significantly different by one-way ANOVA followed by Dunnett’s test (CTL vs A44 or A123, *P* < 0.0001). (F) HEK293T cells were transfected with plasmids encoding mCherry-tagged A44, A123, A171, A44 and A123, or without IBs (negative CTL). At 1 dpt, freshly isolated *E. chaffeensis* was added, and cells were harvested at 2 dpi. Bacterial numbers in each sample were determined by RT-qPCR using primers specific to *Ehrlichia* 16S rRNA and were normalized against the level of human actin mRNA (*ACTB*). Data are presented as the mean ± SD from three independent experiments. *Significantly different by one-way ANOVA followed by Dunnett’s test (CTL vs A44, *P* = 0.0011; CTL vs A123, *P* = 0.0007; CTL vs A44 + A123, *P* = 0.0002)

Secreted native Etf-2 and Etf-2–GFP bind RAB5 on *E. chaffeensis* vacuoles and inhibit dissociation of RAB5 from the vacuole, thereby inhibiting lysosomal fusion [40]. As mCherry-A44 and -A123 inhibited binding between Etf-2C and endogenous RAB5 (Fig. 1B) and inhibited the localization of RAB5 to early endosomes (Figs. 2 and 3), we examined whether mCherry-A44 and -A123 inhibit Etf-2–GFP localization to *E. chaffeensis* vacuoles in RF/6A cells by co-transfection, with mCherry-A171 serving as the negative control. Both A44 and A123, but not A171, significantly inhibited Etf-2–GFP localization to *E. chaffeensis* vacuoles (Fig. 5A–E).

We next examined whether A44 or A123 inhibits *E. chaffeensis* infection and whether there is a synergism between the two IBs. As HEK293T cells can be readily infected with *E. chaffeensis* and their plasmid transfection efficiency is much higher than that of RF/6A cells [40], HEK293T cells were transfected with A44, A123, A44 plus A123, A171, or with no IBs. Bacterial numbers in each sample were determined by RT-qPCR. The results showed that both A44 and A123 significantly inhibited *E. chaffeensis* infection, and the combination of A44 plus A123 was notably more effective than that of either A44 or A123 alone, whereas A171 did not inhibit *E. chaffeensis* infection as compared with the no-IB control (Fig. 5F).

### Involvement of the Arg and/or Gln fingers in the predicted TBC-like motif of Etf-2C in binding to A44 and A123

**Fig. 6:**
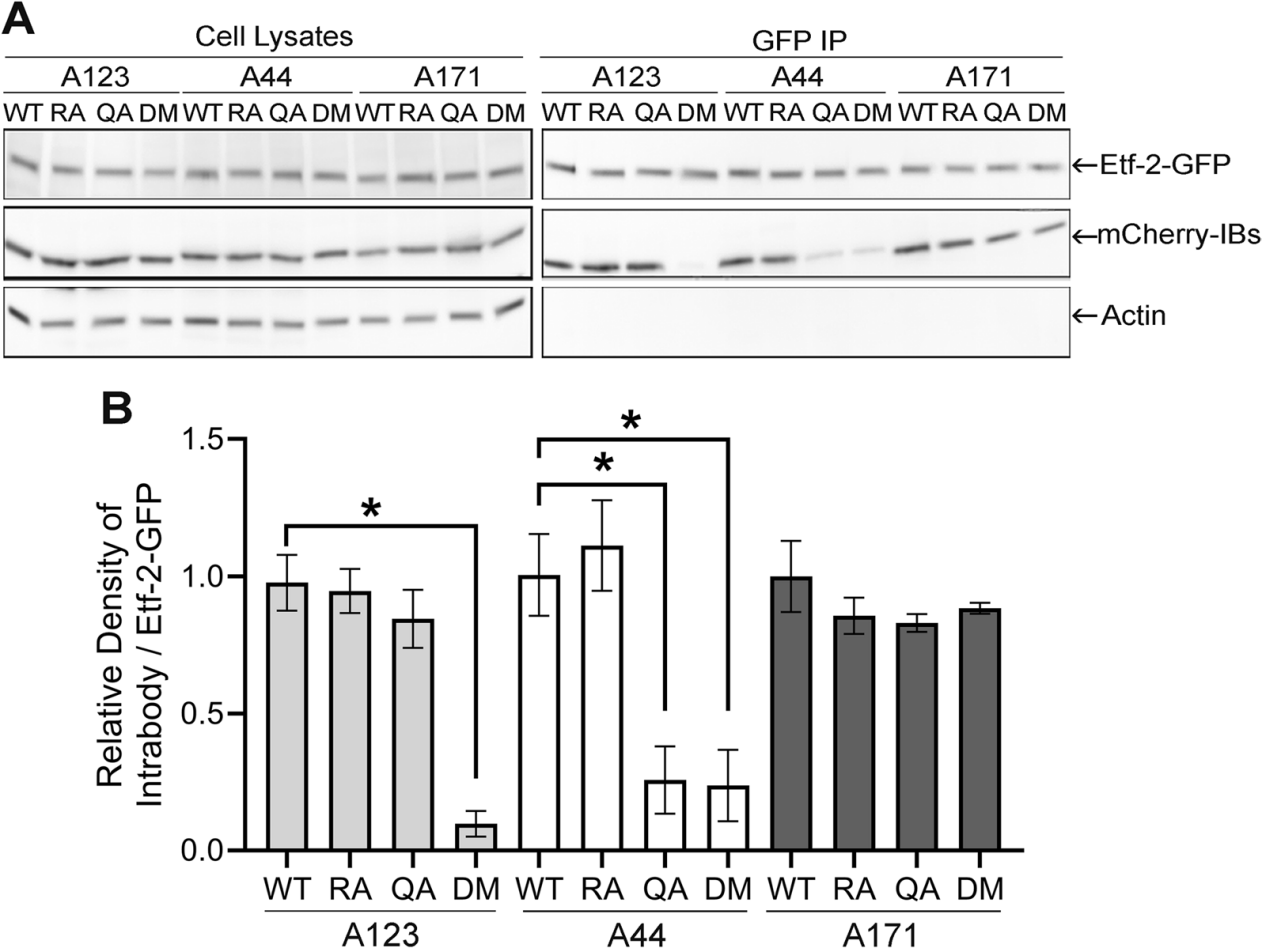
Binding of IBs A44 and A123 depends on the Arg and/or Gln fingers of the predicted TBC-like motif of Etf-2. **A** HEK293T cells were co-transfected with plasmids expressing wild-type Etf-2–GFP (WT) or an Etf-2 mutant (Etf-2^RA^, RA; Etf-2^QA^, QA; Etf-2^RA/QA^, DM) fused with GFP and mCherry-A123, mCherry-A44, or mCherry-A171. The transfected cells were lysed at 2 dpt, and cell lysates were immunoprecipitated with anti-GFP affinity gel. WB was carried out with anti-GFP, anti-mCherry, and anti-Actin. **B** Quantification of binding of the IBs to Etf-2–GFP or its mutants. Data are presented as the mean ± SD from three independent experiments. * Significantly different by one-way ANOVA followed by Dunnett’s test (A123 + WT vs A123 + DM, A44 + WT vs A44 + QA or A44 + DM, *P* < 0.0001)

Although Etf-2 lacks homology to known prokaryotic and eukaryotic GAPs, Etf-2C does contain a predicted TBC-like motif consisting of an Arg finger and a Gln finger from the Tre2-Bub2-Cdc16 (TBC) domain, which is the conserved domain identified in almost all RABGAP proteins [4]. The direct binding of Etf-2 to RAB5-GTP allows it to localize to *Ehrlichia* vacuoles as well as to early endosomes; however, mutation of the Arg or Gln finger of the predicted TBC-like motif in Etf-2 impairs Etf-2 localization to RAB5^CA^ endosomes [40]. To analyze whether binding of IBs to Etf-2 epitopes requires the Arg and/or Gln finger in the predicted TBC-like motif, HEK293T cells were co-transfected with an Etf-2–GFP and an mCherry-IB. The 12 transfections consisted of plasmids expressing GFP fused to wild-type Etf-2 (Etf-2^WT^–GFP), a single Etf-2 mutant—Etf-2^RA^–GFP, Etf-2^QA^–GFP, or Etf-2^RA/QA^ double mutant (Etf-2^DM^–GFP), and mCherry-A123, -A44, or -A171. Anti-GFP affinity pull down and WB results showed that, although binding of A123 to Etf-2^RA^ or Etf-2^QA^ was not reduced as compared with binding to Etf-2^WT^, A123 binding to Etf-2^DM^ was significantly reduced **(**Fig. 6A and B), suggesting that either the Arg or Gln finger is sufficient for binding. In contrast, binding of A44 to Etf-2^QA^ and to Etf-2^DM^ was equally reduced as compared with that to Etf-2^WT^ and to Etf-2^RA^ (Fig. 6A and B), suggesting that only the Gln finger is required for A44 binding. A171 binding to all three Etf-2 mutants was similar to its binding to Etf-2^WT^ (Fig. 6A and B), suggesting that binding of A171 to Etf-2 does not involve the Arg and Gln fingers. Thus, IB binding to the predicted TBC-like motif of Etf-2 may be important for inhibiting Etf-2 functions during *E. chaffeensis* infection.

### Effects of an mRNA-LNP encoding A44 or A123 on *E. chaffeensis* infection

**Fig. 7:**
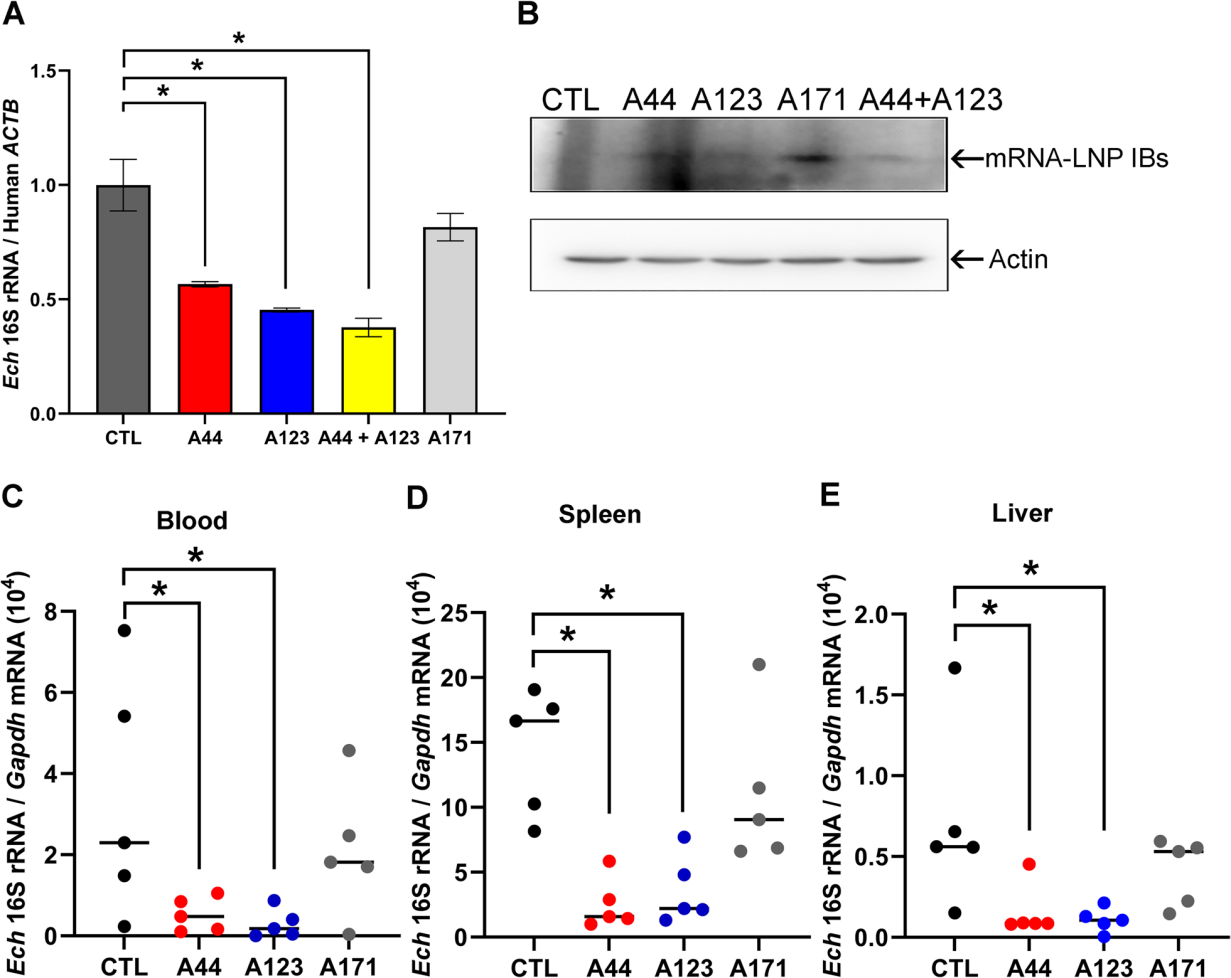
A44 and A123 mRNA-LNPs reduce *E. chaffeensis* infection in HEK293 cells and mice. **A**-**B** HEK293 cells were incubated with LNP-mRNA-IBs or PBS control (CTL) for 6 h and infected with *E. chaffeensis* for 30 h. (A) *Ehrlichia* infection was determined by RT-qPCR analysis using primers specific for *E. chaffeensis* 16S rRNA gene normalized to human *ACTB*. Data are presented as the mean ± SD from three independent experiments. *Significantly different as determined by one-way ANOVA followed by Dunnett’s test (CTL vs A44, *P* = 0.0024; CTL vs A123, *P* = 0.0008; CTL vs A44 + A123, *P* = 0.0004). **B** WB showing expression of IBs using antibodies against HA-tagged IBs and human Actin. **C**-**E** ICR mice were inoculated intraperitoneally with *E. chaffeensis*–infected THP-1 cells that had been preincubated with an mRNA-LNP (A44, A123, or A171) or PBS (CTL) for 12 h. At 1 and 3 d after inoculation, mice were injected intravenously with the same mRNA-LNP or PBS. All mice were euthanized at 5 dpi to collect blood, liver, and spleen samples. **C** Infection with *Ehrlichia* was determined by qPCR of blood samples using primers specific to the *E. chaffeensis* (*Ech*) 16S rRNA gene. Results were normalized based on mouse *Gapdh* expression. **D**-**E** *Ehrlichia* load in spleen (D) and liver (E) samples was estimated by RT-qPCR as in (**A**). The scatter plots show normalized levels in individual mice, with the horizontal bar representing the mean value (*n* = 5). * Significantly different as determined by one-way ANOVA followed by Dunnett’s test with data from blood (CTL vs A44, *P* = 0.0479; CTL vs A123, *P* = 0.0318), spleen (CTL vs A44, *P* = 0.001; CTL vs A123, *P* = 0.0024), and liver (CTL vs A44, *P* = 0.0322; CTL vs A123, *P* = 0.0192)

Although IBs delivered by transfection with plasmids proved useful for in vitro functional analyses, in vivo application requires a different approach. With recent advances in LNP formulations and mRNA engineering, LNP-encapsulated mRNAs can be effectively delivered into many cell types and organs to treat various diseases [14, 18]. In addition, we recently showed that an anti–Etf-3 IB can be delivered by mRNA-LNP [10]. Therefore, we used an mRNA-LNP delivery system for anti–Etf-2 IB expression and examined its effects on *Ehrlichia* infection. HEK293 cells were incubated with A123-, A44-, or A171-encoding mRNA-LNPs for 6 h and then were infected with *E. chaffeensis* for 30 h. RT-qPCR indicated a significant reduction in the bacterial infection of cells incubated with A123 or A44 alone or with A123 and A44, but not with A171 (Fig. 7A). The expression of IBs was detected in cells transfected with all mRNA-LNPs (Fig. 7B).

To examine whether mRNA-LNP IBs can reduce *E. chaffeensis* infection in vivo, the mouse infection model was used [27]. As an obligatory intracellular bacterium, *E. chaffeensis* cannot survive outside the host cell environment; therefore, we inoculated mice with *E. chaffeensis*-infected THP-1 cells instead of purified bacteria. Since *E. chaffeensis*-infected THP-1 cells have already accumulated Etf-2 in the cytoplasm, infected THP-1 cells were preincubated with mRNA-LNPs for 12 h, then inoculated into mice. These mice were further inoculated intravenously with an mRNA-LNP on 1 and 3 d after the initial inoculation. Mice did not show any clinical signs of ehrlichial disease (weight loss, lethargy, anorexia, squinting eyes, or ruffled fur) throughout the 5-day infection period. However, at 5 dpi, *E. chaffeensis* infection was significantly reduced in mice inoculated with A44 or A123 mRNA-LNP, but not with A171 mRNA-LNP or PBS control, as assessed by qPCR analysis of blood samples (Fig. 7C), or by RT-qPCR of spleen and liver samples (Fig. 7D and E).

## Discussion

The present study identified two anti–Etf-2C IBs (A44 and A123) can specifically bind to Etf-2C epitopes containing the predicted TBC-like motif of RABGAP5, which dissociate Etf-2 from RAB5-GTP on early endosomes, thereby facilitating endosome maturation into late endosomes. There was a clear correlation between the inhibition of Etf-2 function and the neutralization of *E. chaffeensis* infection by anti–Etf-2C IBs. Thus, the current study affirmed the previously proposed mechanism of Etf-2 functions, i.e., prevention of maturation of *E. chaffeensis* vacuoles to block lysosomal fusion to facilitate *E. chaffeensis* infection [40]. Two mRNA-LNP encoding anti-Etf-2 IBs significantly inhibited *E. chaffeensis* infection in cell cultures and in a mouse model. Protein expression from mRNA-LNP can occur as little as 1 h when injected into mice [9], thus intravenous inoculation of mRNA-LNP likely contributed to observed effects in mice. In addition, studies showed that protein expression from mRNA-LNP in cell culture occurs as early as 4 h [30], and increases steadily over 12 h [31]. Therefore, pre-treatment with mRNA-LNPs could lead to differences in the viabilities of bacteria in the different inocula, and this could be responsible for or contribute to, the observed differences of *E. chaffeensis* proliferation in mouse blood and tissues at 5-day post inoculation.

Our study showed that a random synthetic VHH library is an effective means of obtaining in vitro and in vivo infection-neutralizing IBs. As compared with previous studies of the immune library of anti–Etf-1 or –Etf-3 IBs obtained by immunization of llamas with recombinant antigens [10, 41], fewer distinct Etf-2–binding VHHs were obtained from the synthetic library, but, similar to the immune library approach, only a fraction of those VHHs were highly expressed in mammalian cells and few were able to effectively inhibit functions of the target antigens. This also underscores the need to have a reliable in vitro assay for functionally screening these libraries, such as the assay developed here using RAB5 pull-down with Etf-2–GFP.

The isolated IBs that inhibited Etf-2 and neutralized *E. chaffeensis* infection (A44 and A123) had a higher binding affinity for Etf-2C than did the non-inhibitory IBs as determined by SPR. In addition, the binding of these IBs involved the predicted TBC-like motif of Etf-2, whereas the non-inhibitory Etf-2-binding IB (A171) did not, suggesting that such binding might contribute to allosteric modulations due to steric hindrance or competitive binding of RAB5-GTP to Etf-2. Interestingly, A44 and A123 IBs bind slightly different epitopes of the predicted TBC-like motif of Etf-2, and we note that the synergistic inhibitory effect between them when delivered through plasmid transfection. Thus, a combination of IBs may improve neutralization efficacy. In addition, some of these VHHs may be useful for studying the three-dimensional structures of Etf-2 and of the anti–Etf-2C VHH complex to improve binding affinity and infection-neutralizing efficacy [2].

IB delivery through mRNA-LNPs is simpler than the method of cell-permeable peptide delivery of IBs, as it does not require recombinant VHH protein preparation and chemical conjugation with cell-permeable peptides and purification [41]. Together with our recent publication [10], the present study supports the mRNA-LNP approach for IB delivery. The loading and composition of the LNPs can influence subsequent expression levels and dynamics [23] and the mechanisms of LNP distribution, metabolism, and elimination in animals are not yet well-understood [39]. Thus, more studies on dosage and uptake, tissue-specific expression, and stability of LNP-mRNAs would help improve the combination of IB mRNA-LNP approach [19, 35, 37].

## Conclusions

Therapeutic options for rickettsia infection, including *E. chaffeensis* infection, are limited to a single broad-spectrum antibiotic. The primary virulence factors of rickettsiae are their abilities to invade and to proliferate rapidly within eukaryotic host cells. Once within their host cells, these intracellular pathogens cannot be targeted by conventional antibodies. However, VHHs can be cloned and expressed intracellularly and are stable in the reduced cytoplasmic environment [6, 13]. In addition, they are advantageous because of their small size and single-domain structure, which allow them to access and bind to cryptic epitopes (regions hidden from larger antibodies). Several VHHs have been approved and are undergoing clinical trials as immunotherapeutics and for the diagnosis of hereditary diseases and cancer [3]. The current study demonstrates the therapeutic potential of IBs for the rickettsial pathogen *E. chaffeensis*.

## Supplementary Information

Additional file 1.

Additional file 2. Plasmid maps of pUC19-IBs and encapsulation efficiency of LNP-mRNA. (A) Schematic illustration of pUC19 plasmid containing IB gene sequences for mRNA synthesis. (B) Encapsulation efficiency of LNP-mRNA determined by the RiboGreen assay and quantified using Cytation 5. Data are presented as the mean ± SD means of each batch (n = 3).

Additional file 3. Colocalization of IBs with Etf-2C–GFP. HeLa cells were co-transfected with plasmids expressing mCherry-tagged IBs and Etf-2C-GFP. At 1 dpt, cells were fixed, and images were acquired by fluorescence microscopy. Scale bar, 10 μm.

## Data Availability

All data are available in the main text and supplementary information.

## Abbreviations

HME: Human monocytic ehrlichiosis
Etf-2: *Ehrlichia* Translocated factor-2
T4SS: Type 4 secretion system
GAP: GTPase-activating protein
VHH: Variable domain of heavy chain of heavy chain only antibody
CDR3: Complementarity-determining region 3
LNP: Lipid nanoparticle
IB: Intrabody
PBS: Dulbecco’s modified phosphate-buffered saline
qPCR: Quantitative polymerase chain reaction
RT-qPCR: Reverse transcription-quantitative polymerase chain reaction
SPR: Surface plasmon resonance
Y2H: Yeast two-hybrid
WB: Western blotting
dpt: Day post transfection
dpi: Day post infection
DIC: Differential interference contrast
MOI: Multiplicity of infection
GFP: Green fluorescent protein
HA: Hemagglutinin
CTL: Control
WT: Wild-type
RA: R188A
QA: Q245A
DM: R188A/Q245A
