# Supplementary material for: Development of mRNA–lipid nanoparticle intrabodies against rickettsial infection
Source: J Biomed Sci. 2025 Aug 12;32:76. doi: 10.1186/s12929-025-01171-5 (PMC12344899; doi:10.1186/s12929-025-01171-5)
Supplement: Supplementary file 1 — Additional file 1. [file 12929_2025_1171_MOESM1_ESM.docx]

# SUPPLEMENTARY TABLE

## TABLE S1. Primers used in this study ^1^

| **Target** | **Primer sequence (5′−3′)** | **References** |
| --- | --- | --- |
| Cloning of Etf-2 and Nbs for *E. coli* expression | | |
| Etf-2C (aa^152–264^) | F: CTAG CCATGG CACTCAAAACATACAAGAGCCAATCAAC  R: TACG CTCGAG TTATCTGCGAAAAAATCTACCACATG | For cloning C-terminal Etf-2 into NcoI (F) and XhoI (R) sites in pET-33b(+) vector. |
| A44-HA-His (codon optimized) | F: GGG CCATGG CAGAAGTACAGCTACAAG  R: GGG GCGGCCGC GCTGGACACGGTAACCTGTG | For cloning A44 into NcoI (F) and NotI (R) sites in pMECS vector. |
| A123-HA-His | F: GGG CCATGG CCCAGCTGCAGGCTTCCG  R: GGG GCGGCCGC GCTACTCACAGTTACCTGCG | For cloning A123 into NcoI (F) and NotI (R) sites in pMECS vector. |
| A171-HA-His | F: GGG CCATGG CCCAGGTGCAGCTGCAGG  R: GGG GCGGCCGC GCTGGACACGGTAACCTGTG | For cloning A171 into NcoI (F) and NotI (R) sites in pMECS vector. |
| Cloning of Etf-2 and Etf-2C Nbs for mammalian expression | | |
| Etf-2-GFP (codon  optimized) | F: GTAC CTCGAG *CGCCACCATGGTG*  CCTACTACACTGGGGACTGTCGCC  R: TACG GGATCC CTCCTGAAGAATCTTCCGCAAGCCTG | (Yan et al. 2018) |
| Etf-2^R188A^-GFP  (codon optimized) | F:AAAAAGCATGGGGATGTGATCGCATTCGACGATGGGTCAGTCCTG  R:CAGGACTGACCCATCGTCGAATGCGATCACATCCCCATGCTTTTT | (Yan et al. 2018) |
| Etf-2^Q245A^-GFP  (codon optimized) | F:GATCTGCTGGACGATTTCGTGGCGTCAGTGGATTACGGGATTGCT  R:AGCAATCCCGTAATCCACTGACGCCACGAAATCGTCCAGCAGATC | (Yan et al. 2018) |
| Etf-2^DM^-GFP  (codon optimized) | F:GATCTGCTGGACGATTTCGTGGCGTCAGTGGATTACGGGATTGCT  R:AGCAATCCCGTAATCCACTGACGCCACGAAATCGTCCAGCAGATC | (Yan et al. 2018) |
| HA-Nbs | F: **GTTCCAGATTACGCT** GAAGACACCATGGCG  R: GTAC GCGGCCGC TCACCTGGCGATGGTTCGGGTCAGAGC  F-overlap: GTAC CTCGAGA *CGCCACCATGGTG* **GTTCCAGATTACGCT** | For cloning HA-Nbs into XhoI (F) and NotI (R) sites on pEGFP-N1 vector. |
| For qPCR and RT-qPCR quantification | | |
| *Ehrlichia* 16S rRNA | F: CGGGGGAAAGATTTATCGCTATTA  R: CGCTTGCCCCCTCCGTATTA | (Yan et al. 2021) |
| Mouse *Gapdh* | F: GTTGTCTCCTGCGACTTCA  R: GGTGGTCCAGGGTTTCTTA | (Miura and Rikihisa 2009) |
| Human *ACTB* | F: AGAGCTACGAGCTGCCTGAC  R: AGCACTGTGTTGGCGTACAG | (Yan et al. 2021) |

__________________

F, forward primer; R, reverse primer; underlined, restriction enzyme sites; bold, modified HA sequence; italicized, Kozak sequences for mammalian cell expression. *ACTB*, β-Actin.

**References:**

Miura K, Rikihisa Y. 2009. Liver transcriptome profiles associated with strain-specific *Ehrlichia chaffeensis*-induced hepatitis in SCID mice. *Infect Immun* **77**: 245-254.

Yan Q, Lin M, Huang W, Teymournejad O, Johnson JM, Hays FA, Liang Z, Li G, Rikihisa Y. 2018. *Ehrlichia* type IV secretion system effector Etf-2 binds to active RAB5 and delays endosome maturation. *Proc Natl Acad Sci U S A* **115**: E8977-E8986.

Yan Q, Zhang W, Lin M, Teymournejad O, Budachetri K, Lakritz J, Rikihisa Y. 2021. Iron robbery by intracellular pathogen via bacterial effector-induced ferritinophagy. *Proc Natl Acad Sci U S A* **118**: e2026598118.
