# Supplementary figures and images for: Development of mRNA–lipid nanoparticle intrabodies against rickettsial infection
Source: J Biomed Sci. 2025 Aug 12;32:76. doi: 10.1186/s12929-025-01171-5 (PMC12344899; doi:10.1186/s12929-025-01171-5)

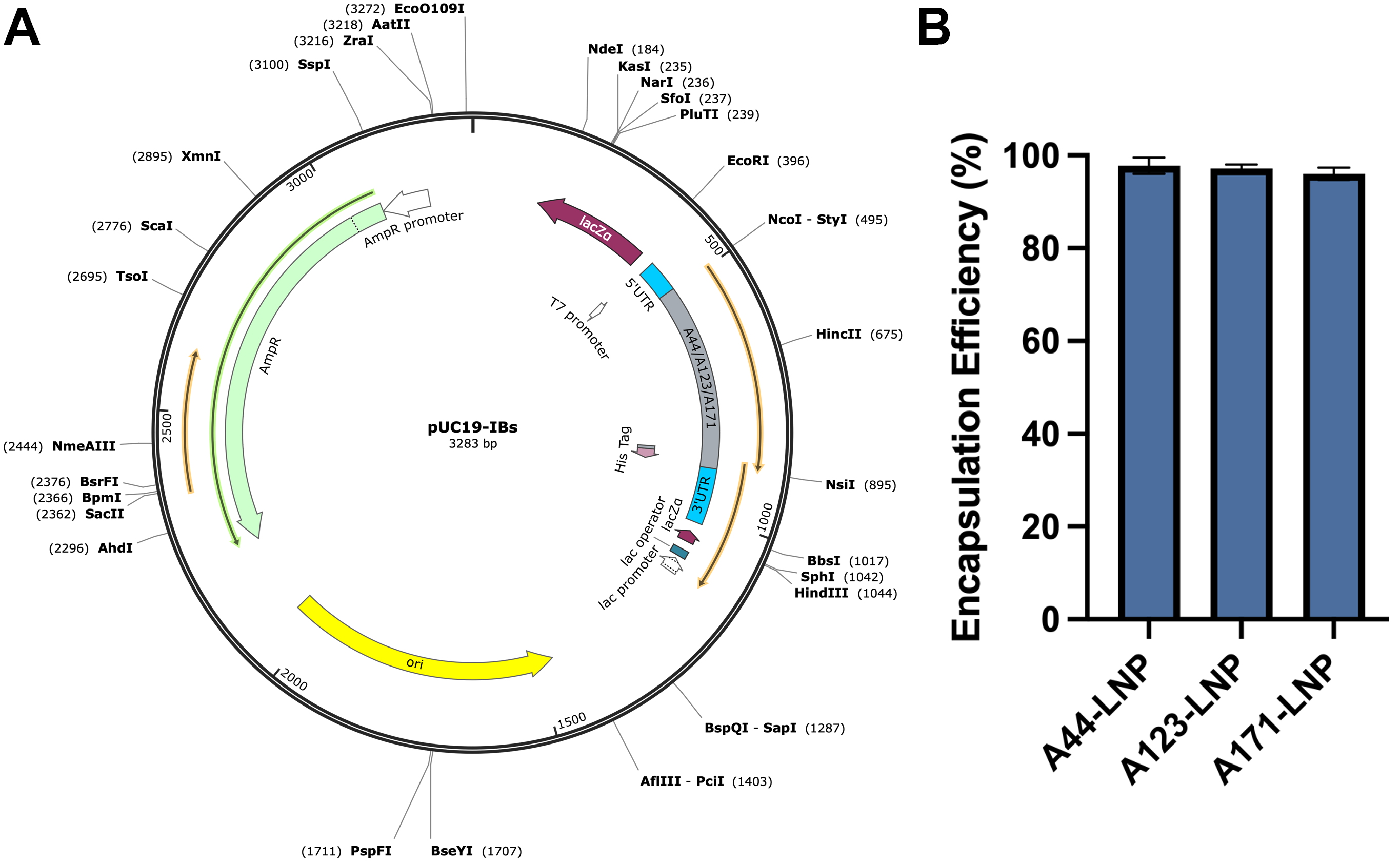

Supplement: Supplementary file 2 — Additional file 2. Plasmid maps of pUC19-IBs and encapsulation efficiency of LNP-mRNA. (A) Schematic illustration of pUC19 plasmid containing IB gene sequences for mRNA synthesis. (B) Encapsulation efficiency of LNP-mRNA determined by the RiboGreen assay and quantified using Cytation 5. Data are presented as the mean ± SD means of each batch (n = 3). [file 12929_2025_1171_MOESM2_ESM.tif]

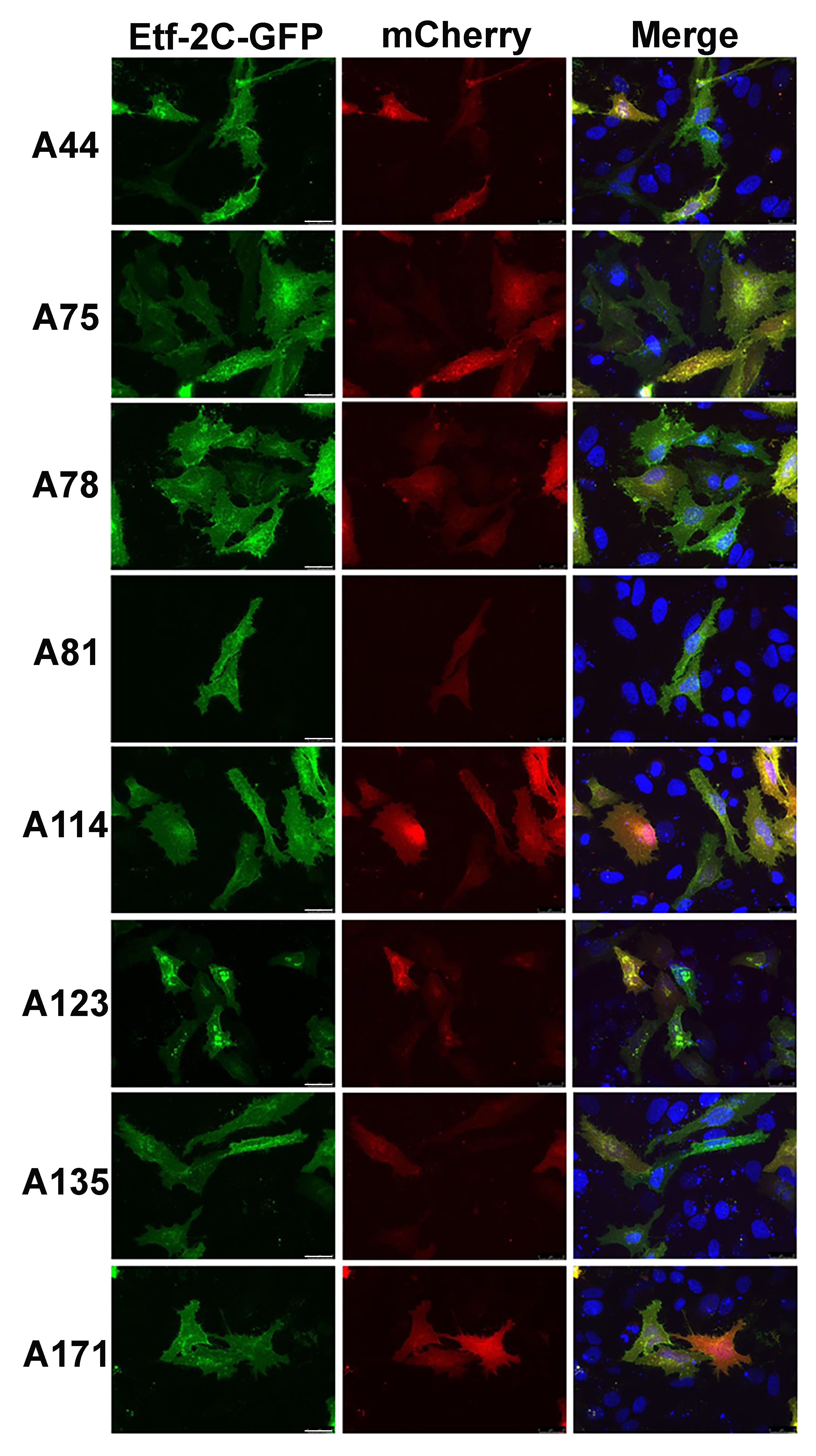

Supplement: Supplementary file 3 — Additional file 3. Colocalization of IBs with Etf-2C–GFP. HeLa cells were co-transfected with plasmids expressing mCherry-tagged IBs and Etf-2C-GFP. At 1 dpt, cells were fixed, and images were acquired by fluorescence microscopy. Scale bar, 10 μm. [file 12929_2025_1171_MOESM3_ESM.tif]
